# Optical Parameters Optimization for All-Time Star Sensor

**DOI:** 10.3390/s19132960

**Published:** 2019-07-04

**Authors:** Wenjie Wang, Xinguo Wei, Jian Li, Jingyuan Du, Guangjun Zhang

**Affiliations:** Key Laboratory of Precision Opto-Mechatronics Technology, Ministry of Education, School of Instrumentation Science and Opto-Electronics Engineering, Beihang University, Beijing 100191, China

**Keywords:** All-Time star sensor, short-wave infrared, optimal detection wavelength band, detection model simulation

## Abstract

As an important development direction of star sensor technology, the All-Time star sensor technology can expand the application of star sensors to flight platforms inside the atmosphere. Due to intense atmospheric background radiation during the daytime, the commonly used star sensors operating in the visible wavelength range are significantly limited in their ability to detect stars, and hence the All-Time star sensor technology which is based on the shortwave infrared (SWIR) imaging system has become an effective research direction. All-Time star sensor detection capability is significantly affected by observation conditions and, therefore, an optimized selection of optical parameters, which mainly includes the field of view (FOV) and the detection wavelength band, can effectively improve the detection performance of All-Time star sensors under harsh observation conditions. This paper uses the model simulation method to analyze and optimize the optical parameters under various observation conditions in a high-altitude environment. A main parameter among those discussed is the analysis of detection band optimization based on the SWIR band. Due to the huge cost constraints of high-altitude experiments, we conducted experiments near the ground to verify the effectiveness of the detection band selection and the correctness of the SWIR star sensor detection model, which thereby proved that the optimization of the optical parameters for high altitudes was effective and could be used as a reference.

## 1. Introduction

Star sensors are one of the most accurate attitude determination instruments to date, and has been widely used in space platforms [1,2,3]. Although the application of star sensors inside the atmosphere is currently difficult, with the rapid development of star sensor technology and the continuous expansion of application fields, the research on All-Time star sensors used inside the atmosphere has become an important development trend. Aircrafts inside the atmosphere such as high-altitude balloons, airships, and high-altitude drones are closer to Earth than low-orbit satellites, and therefore more conductive to providing accurate Earth observation information than satellites. The strong atmospheric background radiation during the daytime is the major factor hindering the application of All-time star sensors [4,5,6,7].

The goal of research on All-Time star sensors is to capture enough stars in the field of view (FOV) in harsh observation environments during the daytime. In order to achieve that goal, it is important and effective to suppress the atmospheric background radiation through optimizing optical design parameters including the detection wavelength band and the FOV.

The study [5] indicates that atmospheric background radiation intensity decreases significantly with the increase of the wavelength. Several research institutions apply spectral filters to filter out the radiant energy of the band below 600 nm based on star sensors operating in the visible wavelength range to accomplish balloon-borne experiments at the height of around 40 km [6,7,8]. However, since atmospheric background radiation increases quickly with the decrease of altitude, the star sensor, correspondingly, cannot perform well at a height below 40 km. In order to better suppress the effects of atmospheric background radiation, a detection system operating around the K-band (2.00–2.32 microns) is an option [7]. However, thermal radiation from the atmosphere and the platform can become a new interference factor; in addition, this kind of infrared detectors need to be cooled to the operating temperature of less than 100 K by specific cooling devices, which greatly increases the difficulty and cost of the system design. Currently, research on daytime star sensors using a SWIR detector operating in the range of 0.9–1.7 um has become a research direction [9]. Based on the SWIR band, through analyzing and determining the optimal detection band, the detection ability of All-Time star sensors can be further improved.

In addition, other optical parameters such the FOV and aperture can also affect the detection capabilities of star sensors. In order to reduce incident sky background radiation and improve the signal-to-noise ratio (SNR) of stars, the existing All-Time star sensors prefer to adopt a small FOV design [7,8]. However, star sensors with a small FOV are designed to point at specific sky areas in the celestial coordinate system and are heavy in weight.

In this paper, firstly, the All-Time star sensor detection model is described, and the difference of factors in the model caused by the selection of SWIR band is analyzed. Then based on the model, optical system parameters including the FOV and the optimal detection wavelength band of All-Time star sensors under different observation environments are determined through a simulation analysis. Lastly, near ground experiments were conducted to validate the model and the effect of the detection band optimization, which proved that the optimization of the optical parameters for the high altitudes is effective.

## 2. All-Time Star Sensor Detection Model

**Figure 1:**
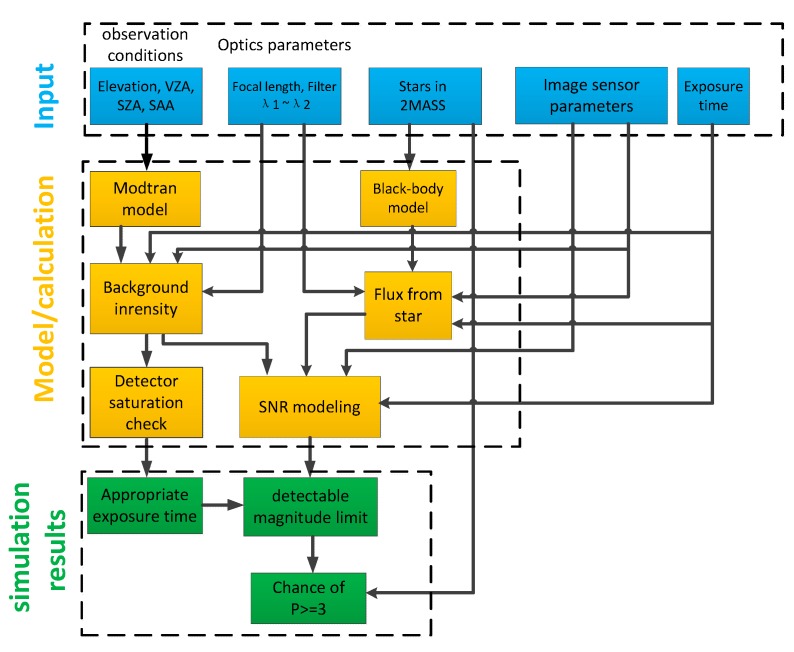
The design flow of All-Time star sensor detection model.

The design flow of the All-Time star sensor detection model is shown in Figure 1. In this section, the All-Time star sensor detection model is described, in which, compared to the visible band, the difference in stellar spectral types of the observed stars due to the selection of SWIR bands is analyzed and the effect of the SWIR detector dark current on the selection of exposure time in a weak atmospheric background radiation environment is also discussed.

Then, the optimal detection wavelength band and other optical parameters of the All-Time star sensor under different observation environments is determined based on this model.

### 2.1. Stellar Flux Estimation by Black-Body Formulation

The spectral energy distribution of stars appears as irregular curves; however, the distribution curves of most stars are close to the black-body radiation curve [10]. Therefore, the star target can be used as black-body to calculate stellar signal energy. The spectral emittance from a black-body is given by Planck’s radiation law as

(1) $$B(\lambda ,T)=\frac{2\cdot \pi \cdot h\cdot c^{2}}{\lambda^{5}\left\{\exp\left[\mathrm{hc}/(k\cdot \lambda \cdot T)\right]-1\right\}}$$

where h = 6.6262 × 10^−34^ J·s, c is the speed of light = 2.997 × 10^8^ m/s, and k is Boltzmann’s constant = 1.381 × 10^−23^ J/k. *λ* denotes wavelength in m and *T* denotes the temperature in Kelvin. According to Equation (1), the radiation spectrum of a star is closely related to the temperature of the surface of the star.

**Figure 2:**
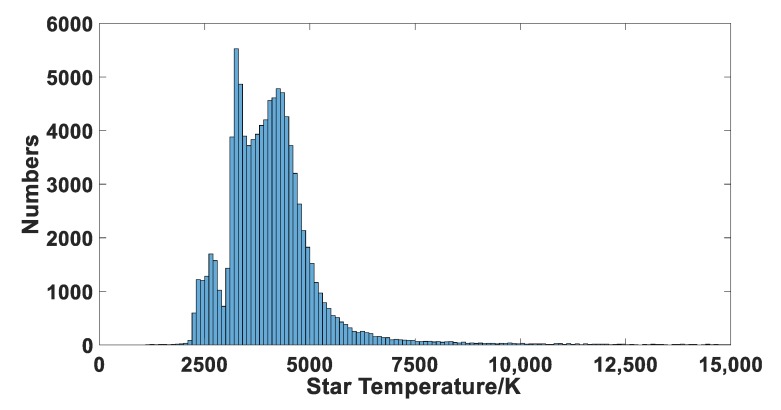
Numbers of stars of different temperatures in 2MASS with magnitudes less than 6.5.

Unlike stars in the visual band, most bright stars at the SWIR band have lower color temperatures. According to the relationship between color index and color temperature in literature [11], a color temperature analysis of stars with instrument magnitudes less than 6.5 in the 2MASS catalog was conducted. The number of stars at different color temperatures is shown in Figure 2. It indicates that the color temperature of about 90% stars is located between 3000 K and 5000 K, which belong to the M, K, and G stellar spectral types.

The number of stellar signal electrons generated on the SWIR detector within a certain exposure time can be expressed as follows.

(2) $$S_{m}=\int_{\lambda_{1}}^{\lambda_{2}}E(m,\lambda ,T)\cdot \tau_{0}(H,\mu ,\lambda )\cdot \frac{\pi D^{2}}{4}\cdot \tau_{opt}(\lambda )\cdot t_{\mathrm{int}}\cdot \frac{1}{W_{ph}}\cdot Q_{0}(\lambda )\cdot d\lambda$$

where *E* (*m*, *λ*, *T*) denotes the energy emitted per second per unit area per wavelength from a star with the absolute surface temperature *T*, and the magnitude m at wavelength *λ*. *τ*_0_ (*H*, *μ*, *λ*) indicates the atmospheric transmittance with the height of *H* and VZA of μ, in which *H* is the height of the star sensor observation position and VZA is the view zenith angles of the star sensor. *D* is the optical aperture, *τ_opt_* (*λ*) is the optical system transmittance, t_int_ is the integration time, $W_{ph}=\frac{\mathrm{hc}}{\lambda }$ is the energy of a single photo, and *Q*_0_ (*λ*) is the quantum efficiency of the SWIR detector.

### 2.2. Atmospheric Background Radiation

The detection capability of All-Time star sensors is significantly affected by changes in the observation conditions. Because of the cost of high-altitude experiments, in order to analyze the detection capability of the star sensor and optimize the optical parameters for different observation conditions in high altitude, it is effective to perform the simulation analysis under different observation conditions using the Modtran software [12,13].

**Figure 3:**
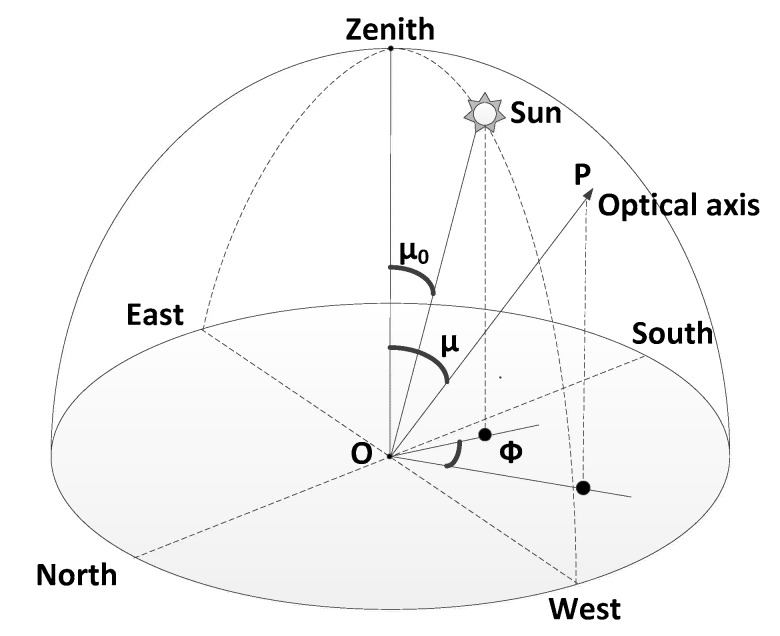
Observation geometry model of the All-Time star sensor.

Figure 3 indicates the observation geometry model of the All-Time star sensor. In the figure below, O is the position of the star sensor, S is the sun, OP is the optic axis, Φ is the view azimuths (SAA), μ is the view zenith angles (VZA), and μ_0_ is the sun zenith angles (SZA).

Considering the atmospheric transmittance, integration time, and parameters of the SWIR detector, the number of electrons generated on the SWIR detector caused by background radiation within a certain exposure time is as follows.

(3) $$S_{bgk}=\int_{\lambda_{1}}^{\lambda_{2}}I_{b}(\lambda ,\mu ,\mu_{0},\phi ,H)\cdot \tau_{0}(H,\mu ,\lambda )\cdot \frac{\pi D^{2}}{4}\cdot \tau_{opt}(\lambda )\cdot t_{\mathrm{int}}\cdot \frac{1}{W_{ph}}\cdot Q_{0}(\lambda )\cdot \Omega d\lambda$$

where Ω is the solid angle (in radian) of one pixel, $I_{b}(\lambda ,\mu ,\mu_{0},\phi ,H)$ is the atmospheric background radiation intensity under specific observation conditions.

### 2.3. SWIR Detector Noise Analysis

InGaAs-based focal plane arrays (FPA) for SWIR detection have broad application and development in space detection and remote sensing [9]. The InGaAs FPA noise mainly includes readout noise, shot noise, and fixed pattern noise, which is dominated by the non-uniformity of FPAs [14].

**Figure 4:**
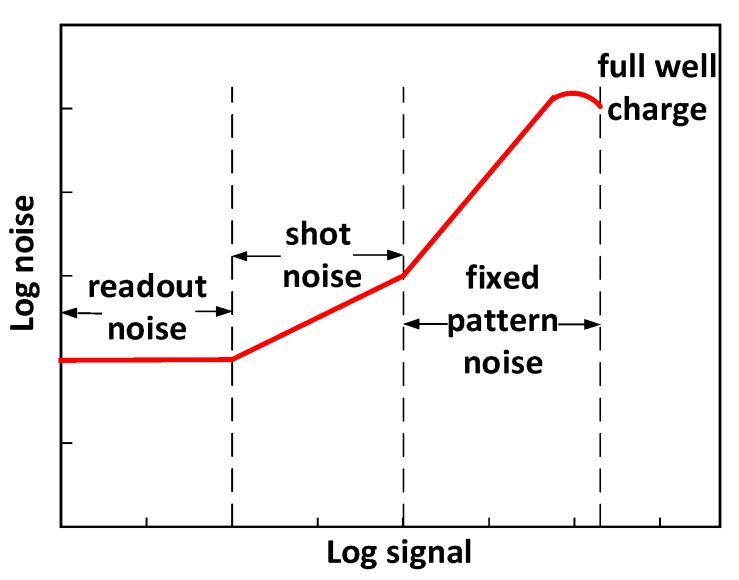
Noise signal transfer curve illustrating three noise regions over the dynamic range.

Figure 4 shows the photon transfer curve (PTC) of a typical SWIR focal plane, which demonstrates the ratio of the number of noise electrons to the number of output signal electrons. The curve shows that the dominant factors of noise signal of the SWIR detector vary at different output signal electrons. When the number of output signal electrons is small, the noise of the SWIR detector is dominated by readout noise, which is mainly due to the readout integrated circuit (ROIC) and is approximated as a constant in the noise model. As the signal electrons increase, the shot noise becomes the major factor, as shown in the second region in the figure above. As the number of signal electrons further increases, the fix-pattern noise, which is mainly due to the non-uniform response of SWIR detector pixels, begins to dominate the detector noise.

The value of FPAs total noise can be given by as follows.

(4) $$\sigma_{total}^{2}=\sigma_{read}^{2}+S+S^{2}\cdot U_{R}^{2}=R+G\cdot t_{\mathrm{int}}+U_{R}^{2}\cdot G^{2}\cdot t_{\mathrm{int}}^{2}$$

where *S* is the number of electrons generated on the SWIR detector pixel, *U_R_* is the fraction of non-uniformity response. The readout noise is defined as a constant *R* and the parameter *G* characterizes the intensity of shot noise.

For the SWIR All-Time star sensor, the shot noise of the SWIR detector includes dark current noise determined by the dark current signal, and the photo-electrical signal shot noise determined by background radiation and the stellar target signal. Under harsh observation conditions, the strong atmospheric background radiation becomes the main factor of parameter *G*.

Different from detectors in the visible range, the dark current signal has a relatively significant effect on the SWIR detector. Even in the case of weak atmospheric background radiation, too-long exposures cannot be used because the large dark current signal can cause the pixels to saturate or the dark current noise will also reduce the star target SNR. Moreover, in general, the fix-pattern noise can be effectively suppressed by the two-point correction method [15], which can be applied on the SWIR star images.

### 2.4. All-Time Star Sensor Detection Model

The star detection sensitivity of the star sensor can be described by the detectable magnitude limit, which can be obtained from the SNR of the star sensor detection system [16,17,18].

Based on the above analysis, the condition that a star spot can be detected is expressed as

(5) $$SNR=\frac{K_{0}\cdot S_{\lim}}{\sqrt{K_{0}\cdot S_{\lim}+S_{bkg}+I_{\mathrm{dark}}\cdot t_{\mathrm{int}}+\sigma_{read}^{2}}}\geq K_{th}$$

where K*_th_* denotes the SNR threshold, which is typically 5, and *S*_lim_ is the number of photoelectrons generated by the weakest detectable star on the detector under certain observation environments. *K*_0_ is defined as the ratio of the star energy on one pixel in the connected components to the total star energy, and this parameter is related to the Gaussian radius of the optical system [19]. The value of *K*_0_ can be analyzed by simulating the energy of the starlight signal as follows.

(6) $$K_{0}=\int_{-1.5}^{-0.5}\int_{-0.5}^{0.5}\frac{1}{2\pi \sigma_{PSF}^{2}}\exp\left[-\frac{x^{2}+y^{2}}{\sigma_{PSF}^{2}}\right]\mathrm{dxdy}=0.1136$$

where *σ_PSF_* is taken as 0.55, and the star spot energy is mainly concentrated in the 3 × 3 pixels.

And then the detectable magnitude limit is as follows.

(7) $$Mv=\log_{2.512}(S_{0}/S_{\lim})=\log_{2.512}(\frac{2K_{0}\cdot S_{0}}{SNR^{2}+\sqrt{SNR^{4}+4\cdot SNR^{2}\cdot (S_{bkg}+I_{\mathrm{dark}}\cdot t_{\mathrm{int}}+\sigma_{read}^{2})}})$$

And *S*_0_ denotes the number of photoelectrons formed by the star with magnitude zero.

Combined with the guide star catalog of SWIR All-Time star sensors [20], the chance of more than three stars being measured in the FOV over the celestial sphere can be obtained and thus the All-Time star sensor detection model is established.

## 3. Optical Parameters Optimization

The established detection model for All-Time star sensors can be used to design optical parameters of All-Time star sensors for different observation conditions through a simulation analysis. Table 1 shows the parameters of the uncooled SWIR detector XFPA-1.7-640-TE1 from XENIC Corporation, which is the SWIR detector chosen for the All-Time star sensor. In the simulation process, in order to prevent the SWIR detector from being easily saturated due to atmospheric background radiation and dark current signals, the exposure time is set at a certain value that the sum of the photoelectrons generated by the these two parts reaches 60% of the well depth of the detector.

For the All-Time star sensor, designing and optimizing the FOV and the detection band play a major role in reducing the atmospheric background radiation and improving the detection capability of the star sensor during the daytime.

### 3.1. The FOV selection for All-Time Star Sensor

Based on the established All-Time star sensor detection model, through simulation, the influence of different FOV selections on the detection sensitivity of the star sensor can be analyzed, so that suitable optical system parameters can be obtained. For the observation height of 20 km with the VZA of 0°, SAA of 180°, and the SZA from 20° to 80°, the analysis of the impact of different FOV selections on detection capabilities is shown in Table 2. In order to ensure excellent image quality and considering the processing difficulty, the relative aperture F = 1.3 was selected.

In Table 2, the chance of more than 3 stars measured in the FOV is taken as evaluation criteria. As can be seen from the table, the detection capabilities of the Ø 4°, Ø 6°, and Ø 8° designs are similar. In order to achieve the miniaturization design goal of the star sensor, the FOV design of Ø 8° is selected. However, Table 2 also indicates that as the SZA gets smaller, the observation conditions become worse and the detection capability correspondingly decreases rapidly. Therefore, based on the 900–1700 nm band, it is possible to improve the performance of the All-Time star sensor through the setting of a suitable spectral filter to operate in the optimal detection wavelength band, in which the background radiation can be further reduced.

### 3.2. The Detection Wavelength Band Optimization

**Figure 5:**
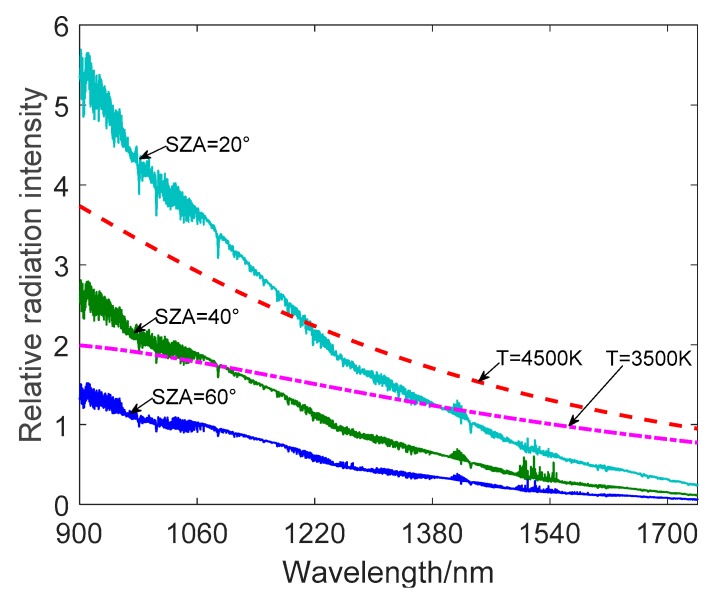
The spectral energy distribution of stars with different color temperatures and atmospheric background radiation at 20 km with different SZA.

Figure 5, which is obtained through a simulation using the Modtran software and Equation (1), shows the spectral energy distribution of stars with different color temperatures and atmospheric background radiation at 20 km with different SZA. It can be seen that atmospheric background radiation decreases as the wavelength increases and the magnitude of the decrease is significantly smaller after around 1200 nm. However, the background radiation between 900 un–1200 nm is not negligible. Therefore, the spectral filtering technique on the basis of the SWIR band can further reduce the influence of atmospheric background radiant energy on the All-Time star sensor. The spectral filtering technique [19] is based on the analysis of the spectral radiation difference between the target and the background. The appropriate spectral filter is then selected to reduce the background radiation photons received by the detector as much as possible while minimizing the attenuation of the stellar target signal.

Meanwhile, the figure above shows that for the All-Time star sensor, the received stellar energy is also reduced after applying the spectral filter. However, the stellar radiation energy decrease is relatively slower as the wavelength changes. Moreover, with the application of a spectral filter, since the atmospheric background intensity is significantly weakened, the exposure time can be appropriately increased, so that the SNR of the star target can be improved.

This section will use the All-Time star sensor detection model established in the previous section to analyze and determine the optimal SWIR detection band under different observation environments. For different optical long-pass filters, the detectable magnitude limits of the All-Time star sensor under different observation environments are simulated.

#### 3.2.1. Optimal Detection Band Selection at Different Observation Heights

**Figure 6:**
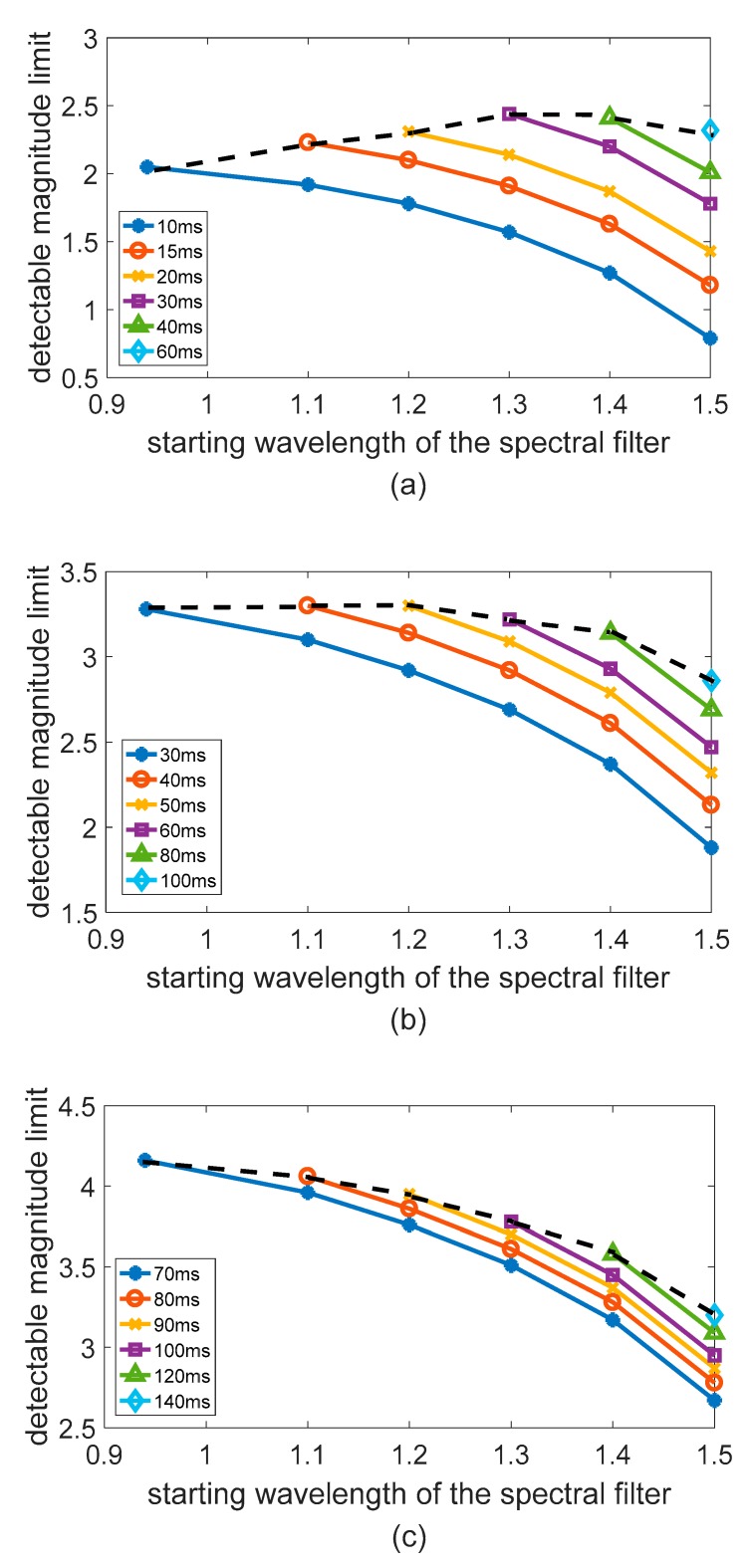
Detectable magnitude limits with different spectral filters at different height; (**a**) 20 km; (**b**) 30 km; (**c**) 40 km.

**Figure 7:**
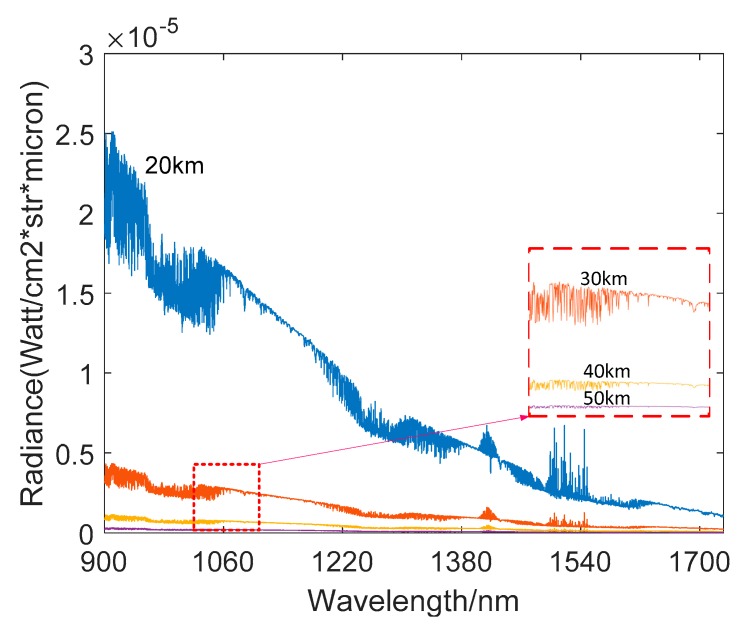
Atmospheric background radiation at 20 km, 30 km, 40 km, and 50 km.

The Figure 6 shows the simulation results obtained by the All-Time star sensor detection model at different observation heights. The VZA was set as 0°, the SZA was 20°, and the SAA was 180°. As can be seen from Figure 6, in the case where exposure time is constant, as the cutoff wavelength of the filter continues to increase, the star sensor’s detection capability decreases accordingly. This is because, although the filter suppresses atmospheric background radiation, part of the energy of the same star is also filtered out. In the case of constant exposure time, the stellar energy reduction is greater than the noise reduction, resulting in the detectable magnitude limit becoming smaller. At the same time, the figure also shows that, under the premise that the number of photoelectrons generated by atmospheric background radiation and dark current signals is constant, the exposure time setting can be correspondingly increased as the cutoff wavelength of the filter increases. The dotted line in the figure shows the highest detectable magnitude limit that can be achieved with different filters. In the Figure 6a, as the cutoff wavelength of the filter becomes larger, the detectable magnitude limit gradually increases and then decreases, reaching its maximum at the cutoff wavelength of 1.3 um. Compared with the case of no filter, the detectable magnitude limit increased by 0.4 and the chance of more than 3 stars measured in the FOV over the celestial sphere increased from 6.3% to 23.18%. At the height of 20 km, the atmospheric background radiation intensity is strong and changes sharply as the wavelength increases. Under this circumstance, the shot noise determined by background radiation dominates the detector noise source. Therefore, when the 1.3 um filter effectively suppresses the background intensity, the exposure time can be increased and the detection capability is significantly improved. Figure 6b shows that the application of the filter has a weak influence on the detection capability at 30 km, while Figure 6c shows that as the filter cut-off wavelength becomes larger, the star sensor’s detection capability becomes weaker at 40 km. This is because the atmospheric intensity at 30 km and 40 km is significantly weaker than that at 20 km and the amplitude of intensity change as the wavelength increases is also significantly smaller, as shown in the Figure 7.

At the height of more than 30 km, as the cut-off wavelength of the filter increases, the radiant energy of the star is suppressed to a greater extent than the suppression of the atmospheric background radiation intensity. Moreover, the exposure time is large under this observation condition, which means that the detector noise is more affected by the dark current. Therefore, even the increase of the exposure time cannot effectively improve the detection capability.

#### 3.2.2. Optimal Detection Band Selection under Different Observation Angles

Even at the height of 20 km, the atmospheric background radiation intensity varies with different observation angles. In order to analyze the influence of the change in observation conditions on the selection of an optimal detection band at 20 km, in the simulation process, the influence of the SZA changes with the VZA of 0° was first studied and then the influence of the SAA changes with the fixed VZA and SZA was analyzed.

**Figure 8:**
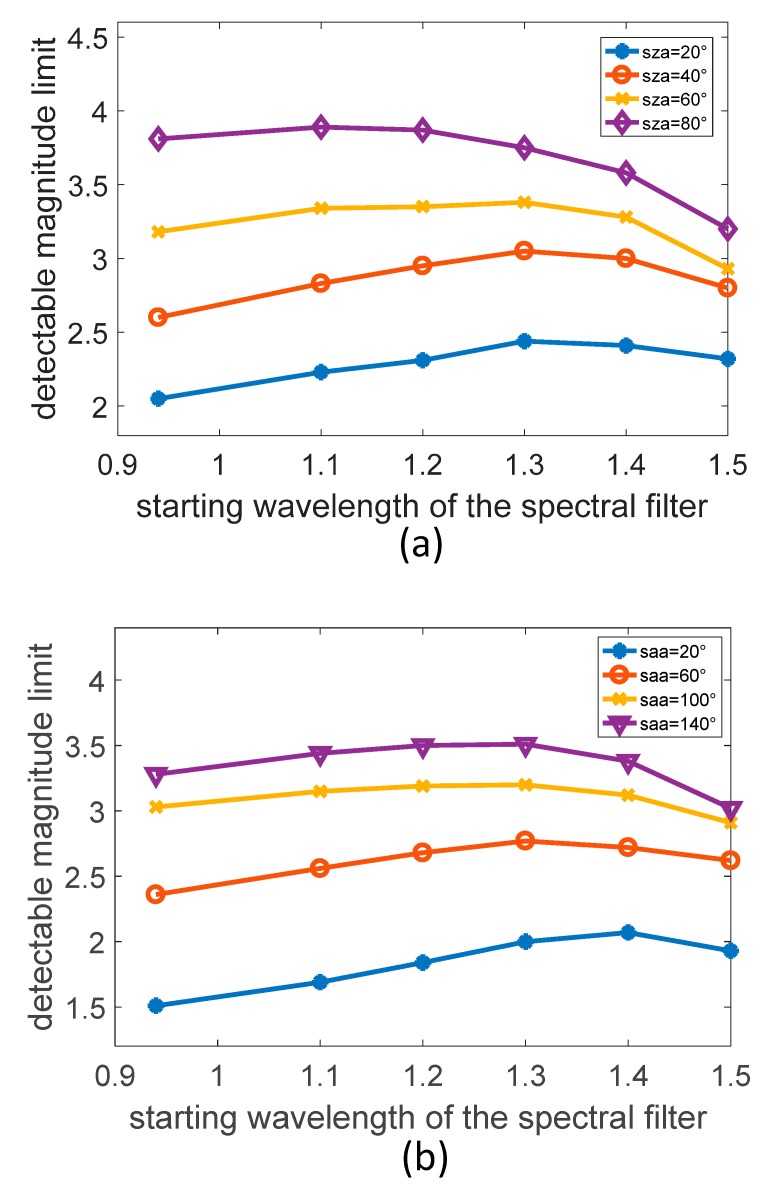
Detectable magnitude limit corresponding to different filters with different observation angles at 20 km. (**a**) SZA changes with VZA of 0° and SAA of 180°. (**b**) SAA changes with VZA of 40° and SZA of 45°.

It can be seen from Figure 8a that in the environment of the height of 20 km, as the SZA becomes larger, the angle between the sun and the boresight direction becomes larger and the atmospheric background intensity gradually becomes smaller. The cutoff wavelength of the optimal filter correspondingly becomes smaller, from 1.3 um to 1.1 um. At the same time, the increase amplitude of the detectable magnitude limit caused by the adoption of the filter is also gradually reduced, from 0.4 to 0.08. In addition, different SAA will also greatly affect the background radiation intensity. It can be seen from the Figure 8b that as the SAA becomes larger, the boresight direction points away from the sun and the background intensity of the atmosphere gradually becomes smaller. The cutoff wavelength of the optimal filter is correspondingly changed from 1.4 um to 1.3 um. In particular, when the SAA is 20°, the boresight direction points close to the sun, and the atmospheric background irradiance is about an order of magnitude higher than the SAA of 140°, therefore, when a filter with a larger cutoff wavelength is used under this condition, the improvement of the detection capability is more remarkable.

Based on the simulation results under different observation conditions at 20 km, we can see that selecting a filter with a cutoff wavelength of 1.3 um can improve the overall detection capability of the star sensor. From the above analysis, it can be concluded that in a high-altitude environment, e.g., when an aircraft is at a low altitude where the atmospheric background radiation intensity is strong, the application of a suitable filter based on a SWIR detector can effectively improve the detection capability of the star sensor. However, as the height of the aircraft flying or residing increases, the application of the filter cannot improve the detection capability and may even weaken it.

## 4. Experiment and Discussion

Based on a SWIR imaging detector, we have developed a prototype of the All-Time star sensor, whose parameters are shown in Table 3. Because the All-Time star sensor prototype is currently difficult to apply to the high-altitude environment around 20 km, experimental data in a high-altitude environment cannot be obtained. Therefore, it is necessary to use experimental data near the ground to verify the previously established model and effect of the detection band selection, which can thereby prove the optical parameters optimization in the previous section.

Combined with currently available experimental conditions, daytime and night experiments were conducted at Xinglong Observatory of National Astronomical Observatories (visibility > 20 km, altitude 960 m). In order to verify the established All-Time star sensor detection model and the effect of detection band selection on the detection capability, the observation results under different observation conditions were analyzed combined with the simulation results of the All-Time star sensor detection model under the same observation conditions.

**Figure 9:**
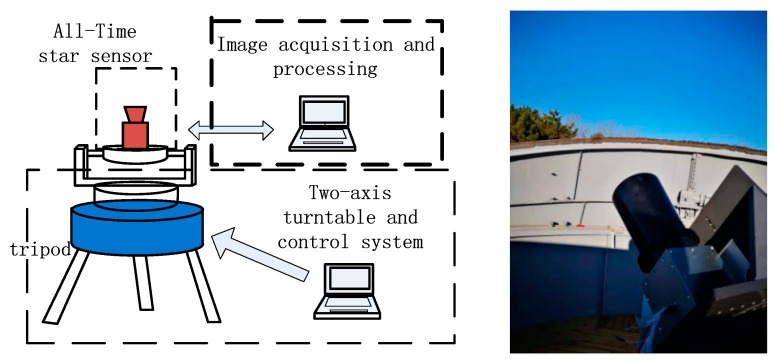
Daytime sky experiment.

The experimental system is mainly composed of three parts: the All-Time star sensor, the image acquisition and processing system, and the turntable and control system, as shown in the Figure 9. The image acquisition and processing system is responsible for the processing and identification of star images, adjusting the integration time of the detection system, and recording the experimental data. During the daytime experiment, the turntable control system is used to guide the boresight direction of the All-Time star sensor to the stars that were planned to be observed.

Because the atmospheric background radiation intensity at the ground level during daytime is much larger than that at the height above 20 km, the detection time was limited to the morning and nightfall when the daytime experiment was conducted at the ground. Meanwhile, considering that the atmospheric transmittance near the ground is close to zero nearby 1.1 um and 1.4 um, the energy of the star near these wavelengths could not reach the All-Time star sensor, so the number of stars available for detection near the ground during daytime was relatively limited.

**Figure 10:**
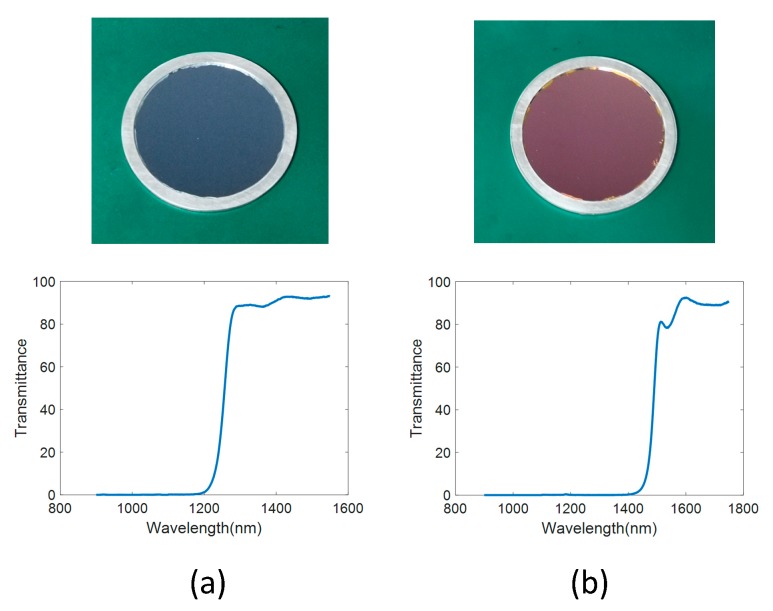
Transmittance of filters (**a**) 1.3 um filter (**b**) 1.5 um filter.

Because of the strong daytime atmospheric background radiation intensity near the ground, it was necessary and effective to use a spectral filter when observing near the ground. Customized bandpass spectral filters were used during the experiments, and the transmittance curve is shown in the Figure 10.

### 4.1. Daytime Tracking Observation Experiment

**Figure 11:**
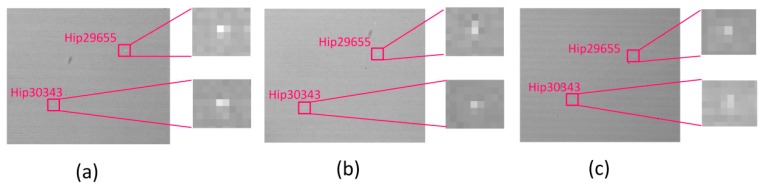
SWIR star images of Hip29655 and Hip30343 captured at different times. (**a**) At 7:00, (**b**) at 7:30, and (**c**) at 8:00.

In order to test the detection capability limit of the All-Time star sensor prototype during the daytime and verify the correctness of the previous established model, the 1.5 um filter was used to continuously track the star HIP29655 (right ascension 95.7401°, declination 22.5136°, instrument magnitude −0.91) from 6:58 am to 8:02 am on 1 November 2018. Figure 11 is the star images taken at 7:00, 7:30, and 8:00 am on 1 November 2018. The two stars in the FOV are Hip29655 and Hip30343, which are marked by red squares. Figure 11 shows that, as the sun continues to rise, the SNR of the stellar target gradually decreases.

According to the observation time, the observation position, and the orientation of the observation target, through calculation, the observation conditions and observation results at 8:00 am on 1 November 2018 are as follows.

From Figure 11 and Table 4, it is shown that the limit conditions for the detection of these two stars were reached at 8:00 am. At this time, after the filter was removed, the detector was rapidly saturated due to strong background radiation, and even if the exposure time was adjusted, the effective detection of the two stars could not be realized, which verifies the necessity and effectiveness of using a filter when the atmospheric background is strong.

**Figure 12:**
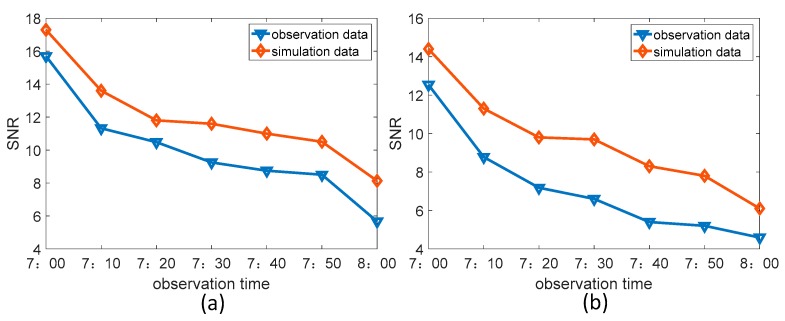
Comparison between the observation results and the simulation results (**a**) Hip29655 (**b**) Hip30343.

The observational conditions, when tracking observations were performed from 7:00 am to 8:00 am, are shown in the Table 5. As the SZA continued to get smaller, which resulted in increasingly harsh observation conditions, the atmospheric background radiation intensity became larger and exposure time was reduced from 45 to 25 ms. The comparison between the observation results of the two stars and their simulation results is shown in Figure 12.

From the comparison results in the above figure, the detection SNRs of the two stars are similar to the simulation results and their trends of change with observation conditions are consistent. As the SZA becomes smaller, the detection SNR becomes smaller. When the SZA reached 76.5°, the detectable conditions limit of the two stars was reached. These tracking observation results can be used to verify the correctness of the All-Time star sensor detection model.

### 4.2. Comparative Experiment with a Different Detection Band

**Figure 13:**
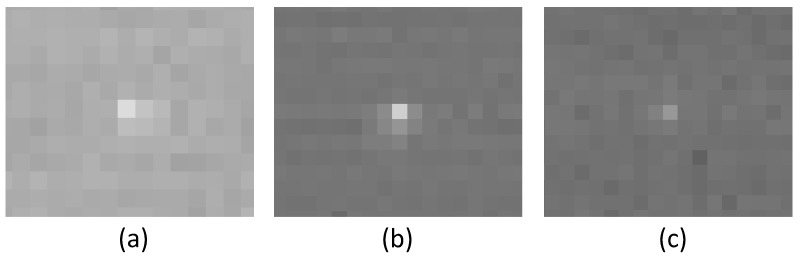
Hip2840 detection results. (**a**) No filter, (**b**) 1.3 um filter, (**c**) 1.5 um filter.

A comparative experiment was conducted to verify the effect of the optimization of the detection band on the observation of the star and the correctness of the model. During daytime, the customized two kinds of filters were used to observe the same star Hip28404 (instrument magnitude 0.25). The detection results are shown in Figure 13.

We compared the observation results with the model simulation results, and the results are as shown in Table 6.

From the comparison results in the table above, the observation SNR is close to the simulated SNR, which can be used to verify the correctness of the previous model and the effect of detection band selection on the detection capability.

**Figure 14:**
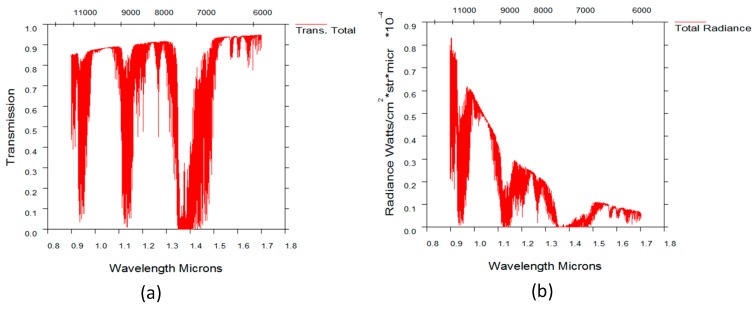
SZA = 89.43°, VZA = 35.3°, SAA = 174.2°. (**a**) Atmospheric transmittance, (**b**) atmospheric background radiation intensity.

Moreover, from the observation results, it can be seen that the 1.3 um filter can further suppress the atmospheric background radiation and improve the SNR of the target detection under the same observation condition. However, when the 1.5 um filter was used, the detection SNR decreased. This is because under this observation condition, the atmospheric background radiation intensity had become relatively small. As shown in the Figure 14, which was obtained through simulation with the Modtran software, the atmospheric background radiation intensity at 1.3 to 1.5 um is at a lower level. At this time, when the 1.5 um filter was used, the suppression of the stellar radiant energy was more than that of the atmospheric background radiation intensity, resulting in a decrease in the SNR.

### 4.3. Detectable Limit Star Observation under Different Observation Conditions

In order to more intuitively verify the detection capability of the detection system and the correctness of the All-Time star sensor detection model, different sky areas at different times were observed through the guidance of the turntable. During the experiments, the detectable limit stars with the SNR close to 5 in the FOV were analyzed and compared with the simulation results of the model under the same observation conditions. And the comparison results are shown in Table 7.

It can be seen from the Table 7 that the observation conditions from right to left were getting worse, the corresponding simulated magnitude limit was gradually smaller, and the observation magnitude limit was similar to it, which can be used to verify the correctness of the detection model.

Since the All-Time star sensor detection model involves many parameters, there was certainly some error between the real observation environment and the atmospheric simulation model Modtran, which leads to a certain degree of error between the observation results and the simulation results. The experimental results show that the established All-Time star sensor detection model can correctly estimate the performance of the All-Time star sensor prototype under various observation conditions. Furthermore, it is proven that the simulation analysis and parameters optimization using the All-Time star sensor detection model are effective and can be used as a reference.

### 4.4. The Chance of More Than 3 Stars Measured in the FOV after the Optimation of the Detection Band

In the near-ground experiments, because the available time for us to observe stars was relatively short, it was difficult to use the prototype to perform detection experiments in a wide range of sky areas to get the chance of more than 3 stars measured in the FOV. Considering that the All-Time star sensor detection model had been verified correctly, the chance of more than 3 stars measured in the FOV of the All-Time star sensor, with 1.3 um filter under different observation conditions at 20 km, was obtained through model simulation to compare with that of the initial star sensor with no filter.

As shown in Table 8, the chance of more than 3 stars measured in the FOV of the All-Time star sensor after the detection band optimization under the observation environment of 20 km was improved significantly overall, especially under the harsh observation conditions. However, even after the detection band optimization, the chance of more than 3 stars measured in the FOV was still insufficient to ensure that the star sensor can continuously output attitude information under the 20 km observation environment. In response to this problem, in the future research, a multi-FOV design will be combined to further improve the chance of more than 3 stars measured in the FOV of the All-Time star sensor during the daytime.

## 5. Conclusions

In this paper, the optical parameters optimization of SWIR All-Time star sensors under different observation conditions in high-altitude was studied. In order to achieve it, the All-Time star sensor detection model was first described, and the difference in the model factors caused by the selection of SWIR band was discussed. Then through using the model simulation method, the corresponding optimal detection bands were analyzed and optimized for various observation conditions. Especially in the case of strong atmospheric background radiation intensity, the optimization of the detection band had a significant improvement on the detection capability of the All-Time star sensor. Finally, through near ground experiments at the Xinglong Observatory of National Astronomical Observatories, the correctness of the All-Time star sensor detection model and the effects of the detection band selection were verified. In this way, it is proven that the parameter optimization of the SWIR All-Time star sensor under different observation conditions at high altitudes can be used as a reference.

## Figures and Tables

**Table 1:** Parameters of the shortwave infrared (SWIR) image sensor.

Parameter	Value
Array format	640 × 512 pixels
Pixel pitch	20 um
Full well charge	800 Ke^−^
Wavelength range	0.94 to 1.7 um
Quantum efficiency	>75%
Dark current	19,000 e^−^/s

**Table 2:** Comparison of detection capabilities for different FOVs of star sensor.

FOV/°	Focal Length/mm	SZA = 20°	SZA = 40°	SZA = 60°	SZA = 80°
Ø 4	180	12.93%	28.2%	58.7%	85.4%
Ø 6	121	9.2%	25.6%	61%	85.6%
Ø 8	92	6.25%	22.3%	62.6%	86.4%
Ø 10	73	3.6%	16.6%	56.4%	86.18%
Ø 12	61	1.89%	11.1%	50.7%	84.5%

**Table 3:** Parameters of the All-Time star sensor.

Parameter	Value
Supply voltage	5 V
Power	6 W
Mass	1.8 kg
FOV	8° × 8°
Optics aperture	70 mm
Focal length	92 mm
Optics penetrating ratio	85%

**Table 4:** Observation conditions and observation results at 8:00 am.

SZA = 76.5° VZA = 54.4° SAA = 148.8°	
**Observed Star**	**SNR**
Hip29655	5.67
Hip30343	4.59

**Table 5:** Observation conditions change over time.

Observation Time	7:00	7:10	7:20	7:30	7:40	7:50	8:00
SZA/°	87.2	85.2	83.4	81.7	80.1	78.4	76.5
VZA/°	43.1	44.9	46.8	48.7	50.6	52.5	54.4
SAA/°	148.5	148.6	148.6	148.9	148.9	148.8	148.8

**Table 6:** Comparison between the observation results and the simulation results.

Observation Condition SZA = 89.13° VZA = 35.3° SAA = 174.2°			
filter	non	1.3 um	1.5 um
Observation SNR	9.7	11.6	6.8
Simulated SNR	11.5	13.4	9.5

**Table 7:** Comparison of observation magnitude limit and simulated magnitude limit under different observation conditions.

Hip Number	Observation Condition	Observation Magnitude Limit	Simulated Magnitude Limit
80704	SZA = 73.5° VZA = 45.3° SAA = 164.2° 1.5 um filter	−0.950	−0.78
29655	SZA = 74.7° VZA=56.2°SAA = 148.8° 1.5 um filter	−0.910	−0.71
30343	SZA = 76.5° VZA = 54.4° SAA = 148.8° 1.5 um filter	−0.720	−0.445
92862	SZA = 86.7° VZA = 8.6° SAA = 14.5° 1.5 um filter	−0.740	−0.36
23015	SZA = 88.0° VZA = 53.7° SAA = 173.5° 1.5 um filter	−0.032	0.049
28404	SZA = 89.1° VZA = 35.3°SAA = 174.2° 1.5 um filter	0.245	0.38
75530	SZA = 90° VZA = 51.9° SAA = 25.5° no filter	0.310	0.54
35601	At night no filter	3.86	3.62

**Table 8:** Chance of more than 3 stars measured in the FOV with different detection bands under different observation conditions at 20 km.

VZA = 0° SZA Varies FOV = 8°				
	20°	40°	60°	80°
No filter	6.3%	22.2%	62.6%	86.4%
1.3 um	23.2%	48.7%	71.8%	85.7%
**VZA = 45° SZA = 40° SAA varies FOV = 8°**				
	**20°**	**60°**	**100°**	**140°**
No filter	1.2%	13.1%	46.25%	62.7%
1.3 um	6.1%	34.53%	58.44%	75.55%
